# Design and 3D Electrical Simulations for a Controllable Equal-Gap Large-Area Silicon Drift Detector

**DOI:** 10.3390/s24051388

**Published:** 2024-02-21

**Authors:** Jun Zhao, Tao Long, Mingyang Wang, Manwen Liu, Minghua Tang, Zheng Li

**Affiliations:** 1 School of Materials Science and Engineering, Xiangtan University, Xiangtan 411105, China; 201610131314@smail.xtu.edu.cn (J.Z.); 201831550096@smail.xtu.edu.cn (T.L.); 202131550147@smail.xtu.edu.cn (M.W.); tangminghua@xtu.edu.cn (M.T.); 2 Institute of Microelectronics, Chinese Academy of Sciences, Beijing 100029, China; 3 College of Physics and Optoelectronic Engineering, Ludong University, Yantai 264025, China; 4 School for Optoelectronic Engineering, Zaozhuang University, Zaozhuang 277160, China

**Keywords:** large-area silicon drift detectors, controllable equal gap, drift channel, low leakage current

## Abstract

In this study, a controllable equal-gap large-area silicon drift detector (L-SDD) is designed. The surface leakage current is reduced by reducing the SiO_2_-Si interface through the new controllable equal-gap design. The design of the equal gap also solves the problem whereby the gap widens due to the larger detector size in the previous SDD design, which leads to a large invalid area of the detector. In this paper, a spiral hexagonal equal-gap L-SDD of 1 cm radius is selected for design calculation, and we implement 3D modeling and simulation of the device. The simulation results show that the internal potential gradient distribution of the L-SDD is uniform and forms a drift electric field, with the direction of electron drift pointing towards the collecting anode. The L-SDD has an excellent electron drift channel inside, and this article also analyzes the electrical performance of the drift channel to verify the correctness of the design method of the L-SDD.

## 1. Introduction

The silicon drift detector (SDD) has extremely broad application prospects in materials analysis [1,2,3,4,5], X-ray fluorescence spectroscopy [6,7], nuclear physics research [8,9,10], medical imaging (such as X-ray CT scanning) [11,12,13], and other fields [14,15]. The L-SDD has a larger sensing area, making it suitable for high-resolution, high-counting-rate X-ray spectroscopic analysis, and has been identified as one of the best detectors for pulsar X-ray detection [16,17].

**Figure 1:**
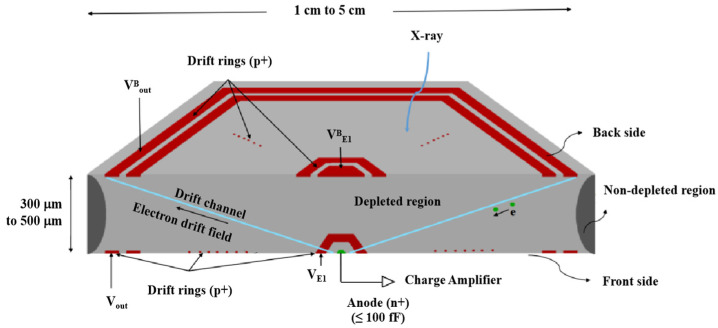
L-SDD structure.

In 1989, E Gatti and P. Rehak invented the hexagonal spiral silicon drift detector [18]. In 2013, Brookhaven National Laboratory carried out in-depth design optimization of the spiral silicon drift detector, which can be generalized to the large-area silicon drift detector [19]. Figure 1 shows the structure of an L-SDD. An L-SDD is mainly composed of an anode, a front drift ring, a back drift ring, and a silicon substrate. An L-SDD requires the design of a double-sided spiral SDD. As the area of the SDD increases, the distance of electron drift also increases. It is necessary to increase the voltage difference on both sides of the L-SDD to provide a sufficient electric field to ensure that the L-SDD has sufficient electron drift velocity. The design of the hexagonal L-SDD allows it to form larger SDD arrays for space applications, such as pulsar navigation.

After appropriate reverse bias voltage is applied to the front and back electrodes, the drift ring provides a potential gradient (or transverse drift field) for the electrons generated by the incident X-ray to drift to the collection anode. Since the collection anode size is independent of the detector size, the L-SDD also has a small collection capacitance while having a large effective detection area [20]. The gap of the SDD is the SiO_2_-Si interface, which has a fixed oxide charge and interface states; this will, in turn, generate a surface leakage current [21]. The spiral ring silicon drift detector designed in this paper can reduce the surface leakage current and improve the detector resolution by controlling the gap of the drift ring.

## 2. Structure Design of the Controllable Equal-Gap L-SDD

**Figure 2:**
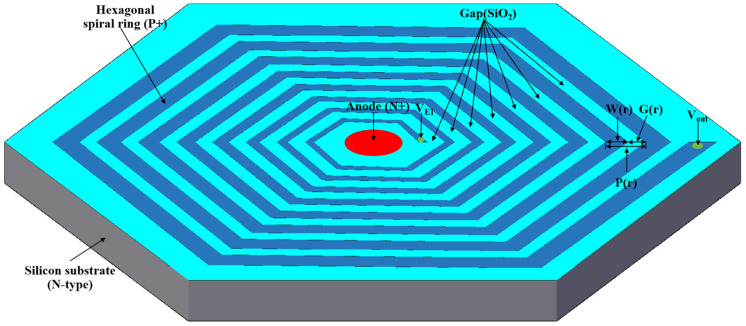
Hexagonal spiral ring L-SDD structure.

The potential distribution inside the SDD is mainly provided by the surface electrodes, so the design core of the L-SDD is the design of the drift rings on the front and back sides. This design uses a spiral hexagon automatic voltage division ring system as the drift rings, as shown in Figure 2. The hexagonal spiral voltage divider ring system is a continuous resistance chain formed by ion implantation. Shown in Equation (1) is the relationship between the spiral pitch, width, and gap, where *W*(*r*) is the width of the spiral ring, *G*(*r*) is the gap between adjacent spiral rings on the front side, and *P*(*r*) is the pitch of the spiral rings at radius *r*:

(1) P(r)=W(r)+G(r)

In an equal-gap design, *G*(*r*) is kept as a constant *G*, and Equation (1) becomes:

(2) P(r)=W(r)+G

The resistance *R*(*r*) of each spiral ring varies with *r*:

(3) $$R(r)=\rho_{S}\alpha r/W(r)$$

where $\rho_{S}$ is the block resistance of the spiral ring ion implantation region, α is determined by the geometry of the spiral cathode ring, the circumference of a circle is equal to αr, and the α of the hexagon is 6.

The voltage difference ΔV(r) between adjacent spiral rings is

(4) ΔV(r)=IR(r)=E(r)P(r)

where *I* is the current of the spiral ring and E(r) is the surface electric field.

According to Equations (3) and (4), the corresponding electric field distribution on the front surface can be obtained as follows:

(5) $$E(r)=\frac{\rho_{S}\alpha rI}{P(r)[P(r)-G]}$$

The surface potential distribution on the front side is obtained by integrating the surface electric field shown in Equation (5):

(6) $$\Phi (r)=\rho_{S}\alpha rI\int_{r_{1}}^{r}\frac{r}{P(r)[P(r)-G]}dr$$

We chose $P(r)\propto r^{\frac{1}{m}}$ dependence here for a slow change in pitch with *r*. But *P*(*r*) can differ depending on the designer’s choice, for example, P(r)∝r or *P*(*r*) = *P*_1_ *=* constant [19]. In this design, the relationship between the pitch of the front spiral cathode ring *P*(*r*) and *r* is chosen as follows:

(7) $$P(r)=P_{1}{\left(\frac{r}{r_{1}}\right)}^{\frac{1}{m}}\;(m\;\mathrm{is}\;a\;\mathrm{real}\;\mathrm{number})$$

where *P*_1_ is the pitch of the first spiral ring, *r*_1_ is the radius of the first spiral ring, and different *P*_1_ values can be obtained by adjusting the value of *m*.

In this design, the relationship between *G* and *P*_1_ is chosen as follows:

(8) $$G=kP_{1}\;(0<k<1)$$

*G* is controlled by *k* and *P*_1_, and *k* = 0.7 is selected in this design as a practical number in accordance with the actual process.

If we substitute Equation (7) into Equation (6), and let $x={\left(\frac{r}{r_{1}}\right)}^{\frac{1}{m}}$, we obtain $dr=mr_{1}x^{m-1}dx$, and the front surface potential distribution:

(9) $$\Phi (r)=\frac{m\rho_{S}\alpha r_{1}^{2}I}{P_{1}^{2}}\int_{r_{1}}^{{\left(\frac{r}{r_{1}}\right)}^{\frac{1}{m}}}\frac{x^{2(m-1)}}{x-k}dx$$

The back surface potential distribution Ψ(r) is chosen to be related to the front surface potential distribution as follows:

(10) $$\Psi (r)=V_{E1}^{B}+\gamma \Phi (r)\;(0<\gamma <1)$$

where *γ* affects the drift channel position, the selection of 0.7676 is good in this design [19], and $V_{E1}^{B}$ is the voltage of the first ring of the back spiral rings, which is chosen as follows:

(11) $$V_{E1}^{B}=V_{fd}+\gamma V_{E1}$$

where $V_{E1}$ is the voltage of the first ring of the front spiral rings, and $V_{fd}$ is the full depletion voltage:

(12) $$V_{fd}=qN_{D}d^{2}/2\varepsilon_{0}\varepsilon_{si}$$

$N_{D}$ is the doping concentration of the silicon substrate, $\varepsilon_{si}$ is the dielectric constant of silicon, $\varepsilon_{0}$ is the dielectric constant of the vacuum, and d is the thickness of the detector substrate.

Combining Equations (9) and (10), the back surface potential distribution is obtained:

(13) $$\Psi (r)=V_{B}+\gamma \frac{m\rho_{S}\alpha r_{1}^{2}I}{P_{1}^{2}}\int_{r_{1}}^{{\left(\frac{r}{r_{1}}\right)}^{\frac{1}{m}}}\frac{x^{2(m-1)}}{x-k}dx$$

The relationship between the first ring pitch $P_{1}^{B}$ of the back spiral ring and *P*_1_ is as follows:

(14) $$P_{1}^{B}=\frac{P_{1}}{\sqrt{\gamma }}$$

The back spiral ring pitch $P^{B}(r)$ is as follows:

(15) $$P^{B}(r)=P_{1}^{B}{\left(\frac{r^{B}}{{r_{1}}^{B}}\right)}^{\frac{1}{m}}$$

The radius of the spiral ring increases from *r_n−_*_1_ to *r_n_* for one complete rotation, i.e., at any radius *r*, for a rotation angle θ(r) increasing by 2π, the spiral ring radius *r* increases by one pitch *P*(*r*). We thus have dθ(r)/2π=dr/P(r) and

(16) $$\theta (r)=\int_{r_{1}}^{r}\frac{2\pi }{P(r)}dr$$

θ increases by an angle of $\frac{\pi }{3}$ for each vertex point when it is a hexagon. Thus, by obtaining the data of each vertex point of the spiral ring, we can easily design the hexagonal spiral rings.

The thickness of the silicon substrate of the SDD is set to *d* = 500 µm, with light n-type doping of 4 × 10^11^/cm^3^. The anode is heavily n-type doped at 1 × 10^19^/cm^3^ using phosphorous ion implantation. The spiral rings on the front and back surfaces are heavily p-type doped at 1 × 10^19^/cm^3^ with boron ion implantation. The radius of the anode is 180 µm, and the radius of the outmost ring of the spiral rings on both the front and back sides is *R* = 10,000 µm. The radius of the first spiral ring on the front side is *r*_1_ = 200 µm. The other settings are as follows: 0.7676 for γ, 1.5 for *m*, and 0.7 for *k*. The block resistance $\rho_{S}$ is 2000 Ω, and *I* is 20 × 10^−6^ A. The voltage on the first ring of the front spiral ring $V_{E1}$ is −5 V, the voltage on the outmost ring of the front spiral ring $V_{out}$ is −300 V, the voltage on the first ring of the back spiral ring $V_{E1}^{B}$ is −80 V, and the voltage on the outmost ring of the back spiral ring $V_{out}^{B}$ is −310 V.

**Figure 3:**
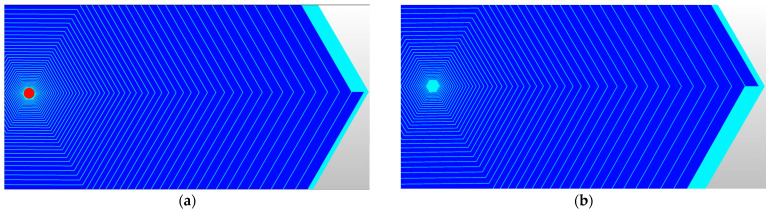
L-SDD spiral ring design: (**a**) front-surface spiral ring with 59 rings; (**b**) back-surface spiral ring with 50 rings.

*P*_1_ is obtained by Formula (9) using boundary conditions of voltages on the first ring $\Phi (r_{1})=V_{E1}$, $\Phi (R)=V_{out}$, and the relationship between *P*(*r*), *W*(*r*), and *r* for each spiral ring is obtained by Formula (6), and the spiral ring design is obtained using Equation (16), with the results shown in Figure 3. Figure 3a shows the design of the front-surface spiral ring with 59 rings, and the red circle is the collecting anode. Figure 3b shows the design of the back-surface spiral ring with 50 rings.

**Figure 4:**
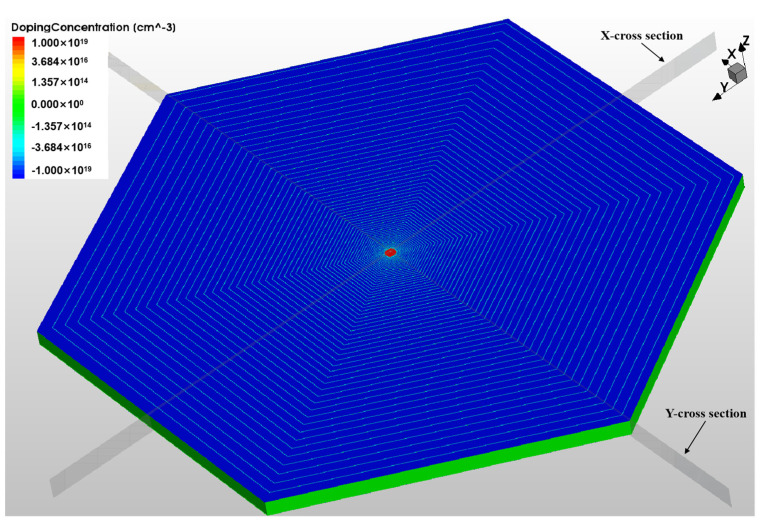
Three-dimensional model of the L-SDD.

In the actual design, the last spiral ring is designed as a closed ring, forming a part of the outermost boundary to reduce the high field effect of the boundary, as shown in Figure 4.

## 3. ThreeDimensional Electrical Simulation of LSDD

### 3.1. Electrical Potential Distribution of LSDD

**Figure 5:**
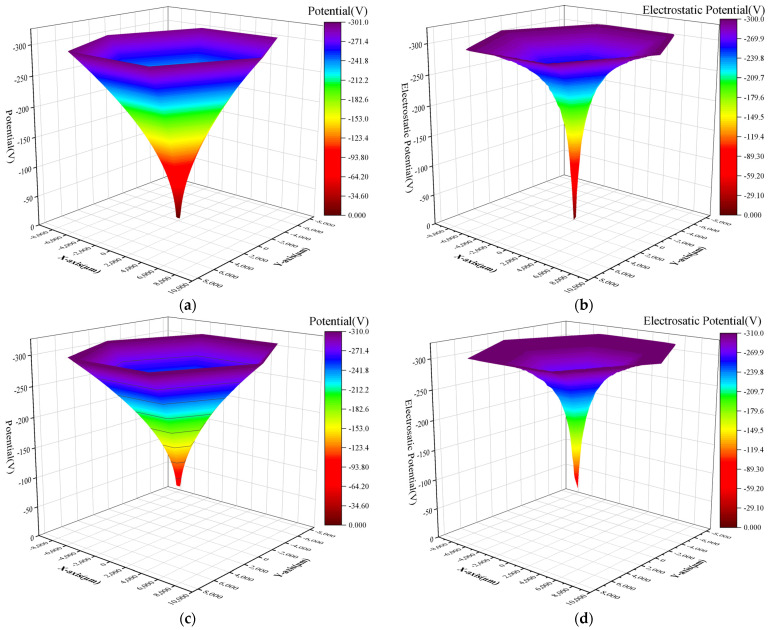
Surface potential distribution of L-SDD: (**a**) designed front surface potential; (**b**) simulated front surface potential; (**c**) designed back surface potential; (**d**) simulated back surface potential.

Figure 5a,c were obtained by solving Equations (9) and (13), and Figure 5b,d were obtained using 3D TCAD simulation of the detector’s front and back surface potential distribution. It can be found that the TCAD-simulated surface potential distribution has a similar trend to the designed surface potential distribution. Since the design is a two-dimensional calculation and the simulation is a three-dimensional calculation, the simulation in the region where the spiral ring is more dense (the central region) has a larger potential gradient than the design.

We cut planes in the Y and X cross-sections at the center of the detector to obtain the internal potential distribution of the detector.

**Figure 6:**
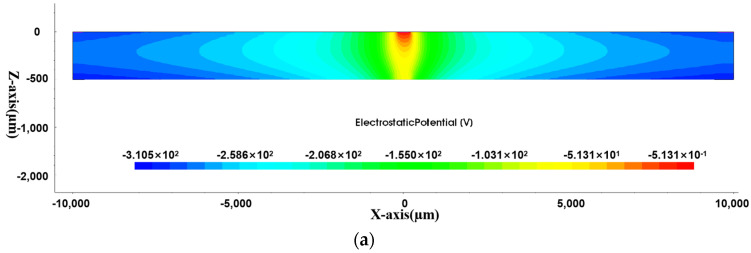

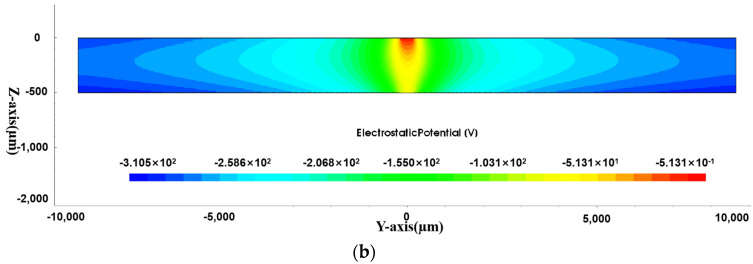
Potential distribution in L-SDD: (**a**) potential distribution in Y cross-section; (**b**) potential distribution in X cross-section.

From Figure 6, it can be seen that the potential of the anode is 0, and the negative potential inside the detector is uniformly and symmetrically distributed around the anode. The potential distribution on the X cross-section is similar to that on the Y cross-section. In order to further study the internal potential of the detector, we extracted the potential on the Y cross-section and created a 3D drawing to better visualize the potential distribution inside the detector.

**Figure 7:**
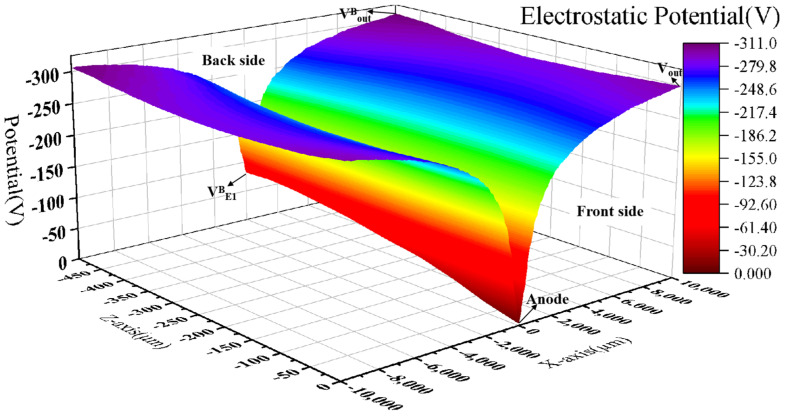
Three-dimensional potential distribution in Y cross-section.

As can be seen from Figure 7, a continuous potential distribution is formed inside the detector by applying a suitable voltage to the electrode. The negative potential is symmetrically distributed around the anode, which is the lowest potential inside the detector. The reasonable potential distribution of the detector forms a good electric field distribution inside the detector.

### 3.2. Electric Field Distribution in L-SDD

**Figure 8:**
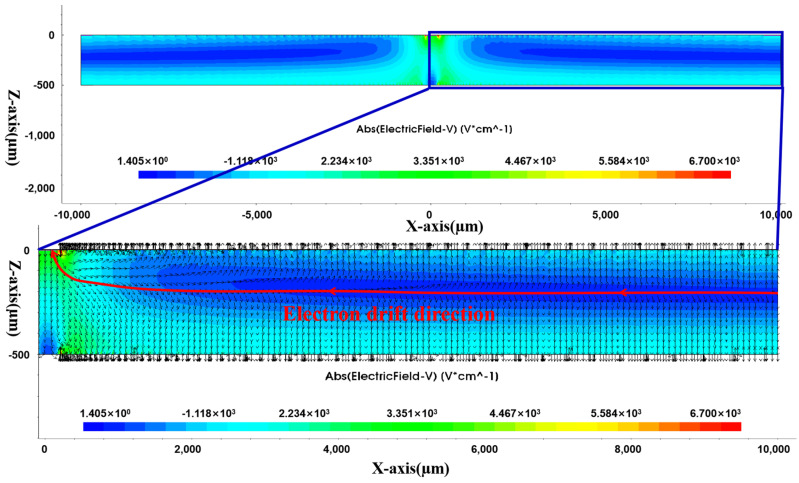
Electric field distribution in Y cross-section.

Figure 8 shows the electric field distribution in the Y cross-section of the detector. There is a linear low-electric-field region in the detector, and the low-electric-field region gradually approaches the middle of the Z-axis (detector thickness) with the increase in the radius. This low-electric-field region is the transverse-drift-electric-field region of electrons. We enlarge the electric field area on the right side of Figure 8 and display the direction of the electric field vector. The opposite direction of the electric field vector arrow indicates the direction of electron drift. From the red arrow, it can be seen that the direction of electron drift ultimately points towards the anode.

### 3.3. Electron Concentration Distribution in L-SDD

**Figure 9:**
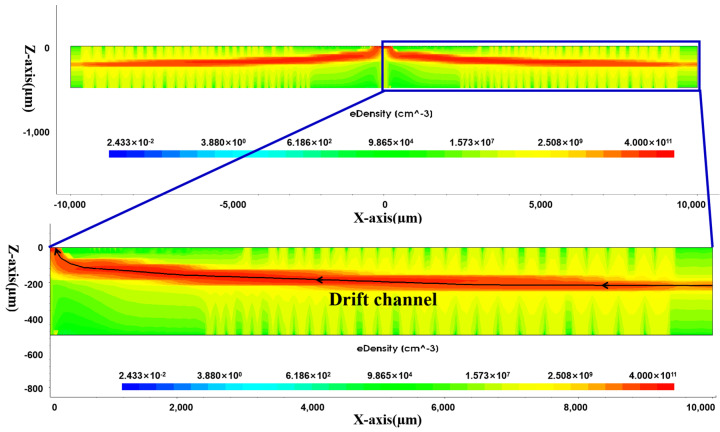
Distribution of electron concentration in the Y cross-section.

Figure 9 shows the electron concentration distribution on the right side of the detector Y cross-section, which is consistent with the prediction made by the electric field distribution. The electrons in the detector first drift towards the drift channel, and then, drift towards the anode to be collected, forming a drift channel, as indicated by the red belt in the electron concentration distribution in the middle of the detector Y cross-section. This drift channel can be designed to be a larger SDD; even if an SDD with a radius of 2–5 cm is designed, the drift channel is also inside the detector.

Regarding the total leakage current of the detector *I_leakcurrent_ = I_s_ + I_b_*, *I_s_* is the surface leakage current, which is proportional to the surface area of the thermal oxide. *I_b_* is the bulk leakage current that is dominated by the bulk leakage current that is proportional to the depleted volume of the detector, since under the reverse bias and full depletion conditions, the junction diffusion current is negligible. Under this full depletion condition, the bulk leakage current is proportional to the entire bulk of the detector, which is the detector area *A* times its thickness *d*, which is independent of *W*(*r*), and is a constant given the same detector materials. From Figure 9, it can be seen that the detector bulk has been fully depleted. Therefore, under this full depletion condition, our design of a reduced Si-SiO_2_ surface area will effectively reduce the total detector leakage current.

### 3.4. Analysis of the Electrical Performance of Drift Channels

To verify the correctness of the device simulation and design, the potential and electric field data in the simulated drift channel are extracted and compared with the design data.

The designed potential in the drift channel (drift potential) $\Phi (r,x_{ch})$ [20] is

(17) $$\Phi (r,x_{ch})=\frac{qN_{D}}{2\varepsilon_{0}\varepsilon }x_{ch}{\left(r\right)}^{2}+(\frac{\Psi (r)-\Phi (r)}{d}-\frac{qN_{D}d}{2\varepsilon_{0}\varepsilon })x_{ch}(r)+\Phi (r)\;$$

And the designed electric field in the drift channel (drift field) $E_{dr,r}$ [20] is

(18) $$E_{dr,r}=\frac{\partial \Phi (r,x_{ch}(r))}{\partial r}=\frac{1}{2}(\frac{d\Psi (r)}{dr}+\frac{d\Phi (r)}{dr})-\frac{1}{2}[\frac{\left(\Psi (r)-\Phi (r)\right)}{V_{fd}}](\frac{d\Psi (r)}{dr}-\frac{d\Phi (r)}{dr})$$

**Figure 10:**
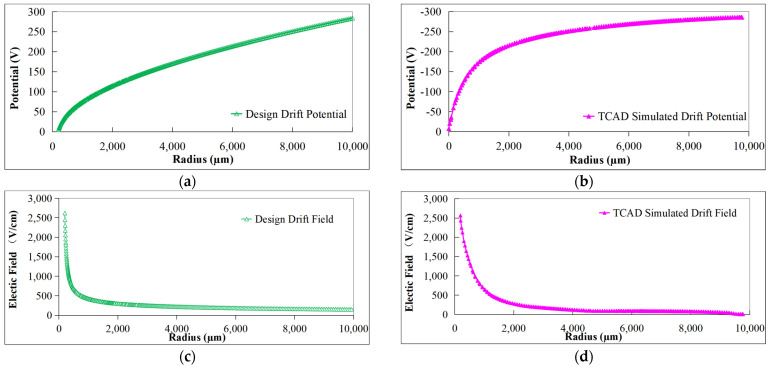
L-SDD drift potential and drift field: (**a**) designed drift potential; (**b**) simulated drift potential; (**c**) designed drift field; (**d**) simulated drift field.

Figure 10a,c show the designed drift potential distribution and drift electric field distribution obtained through Equations (17) and (18). Figure 10b,d extract the drift potential distribution and drift electric field distribution of the drift channel in Figure 9. It can be found that the TCAD simulated drift potential and drift field have a similar trend to the designed drift potential and drift field. Since the design is a 2D calculation and the simulation is a 3D calculation, the simulation in the region where the spiral ring is more dense (the central region) has a larger drift potential gradient than the design. According to the simulation results, the drift field in most of drift channel region is more than 100 V/cm, which can provide a large enough drift field for electron movement.

By using the drift field, the drift time $t_{dr}$ of electrons at different positions in the detector can be predicted based on the electron drift formula:

(19) $$t_{dr}=\int_{r_{1}}^{r}\frac{1}{\mu E_{dr,r}}dr$$

Here, *µ* is the electron drift velocity.

**Figure 11:**
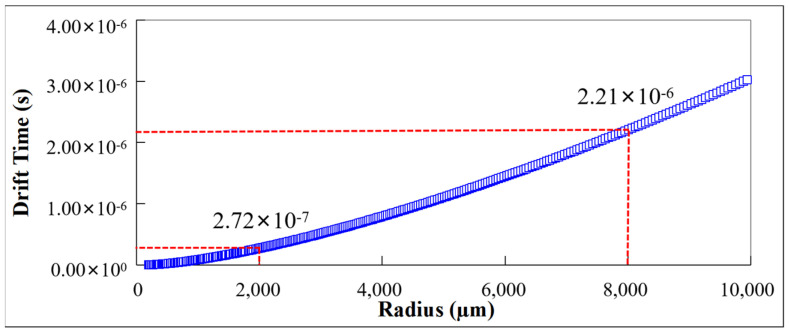
Drift time in L-SDD.

The drift time of electrons is influenced by the drift channel path and drift electric field. The L-SDD designed in this paper has a good drift channel path and provides a drift electric field larger than 100 V/cm, which gives a near-minimum drift time of electrons. We used Equation (19) to calculate the electron drift time of the detector at different positions, as shown in Figure 11. We can simulate heavy ion incidence at different positions of the detector and verify the electron drift time in Figure 11 based on the collection time of the induced current.

**Figure 12:**
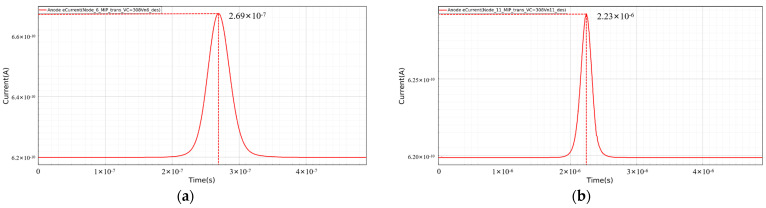
Induced current generated by heavy ion incidence in L-SDD: (**a**) induced current at *r* = 2000 µm; (**b**) induced current at *r* = 8000 µm.

Figure 12 shows the induced current generated by the simulated heavy ion incident on the L-SDD at positions *r* = 2000 µm and *r* = 8000 µm on the incident surface (back side). The time for the detector to collect the induced current at position *r* = 2000 µm is 2.69 × 10^−7^ s (Figure 12a), and the time for the detector to collect the induced current at position *r* = 8000 µm is 2.23 × 10^−6^ s (Figure 12b), which is consistent with the corresponding electron drift time predicted in Figure 11.

**Figure 13:**
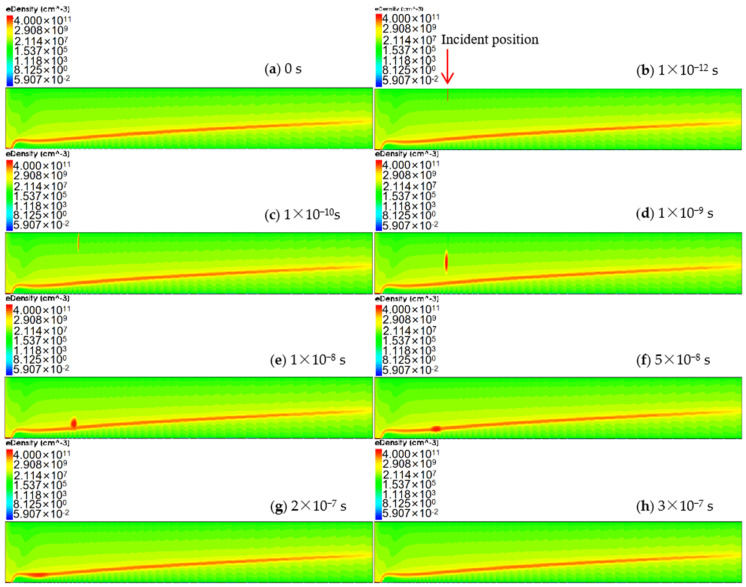
The drift process of charge carriers (electrons) over time at position *r* = 2000 µm.

Figure 13 shows the continuous variation in electron concentration after the heavy ion incident on the back side of the L-SDD at *r* = 2000 µm. At a time of 1 × 10^−12^ s, a beam of heavy ion incident at a position of *r* = 2000 µm, and a small high-electron-concentration region (an electron cloud) can be observed to be formed in the detector (marked by the red arrow in Figure 13b). This electron cloud continues to expand while moving towards the drift channel. At a time of 5 × 10^−8^ s, as shown in Figure 13f, it can be seen that this electron cloud has entered the drift channel and moved towards the anode. Finally, as shown in Figure 13h, at a time of 3 × 10^−7^ s, the electron cloud disappears, and all electrons have drifted to the anode and been collected, restoring the same state as before the heavy ion incident (Figure 13a).

## 4. Conclusions

In this work, we designed an equal-gap large-area spiral ring silicon drift detector with a radius of 1 cm, completed a 3D model and electrical characteristic simulation of the L-SDD, and determined the electronic drift channel inside the L-SDD based on the 3D potential distribution, electric field vector, and electron concentration profile. This design gives a drift channel close to a straight line inside the L-SDD. This paper also compares the simulation and design results of the potential distribution and electric field distribution of the drift channel.

The simulation results have the same trend as the design results. The drift field in the drift channel region is more than 100 V/cm, which provides a large enough drift electric field for electron movement. The drift time of electrons in the detector is calculated according to the drift field, and the maximum drift time of electrons is 3.05 × 10^−6^ s. In addition, a heavy ion incident simulation is carried out at *r* = 2000 µm and *r* = 8000 µm of L-SDD, and the results verify the electron drift path and electron drift time. This shows that the controllable equal-gap spiral ring L-SDD designed in this work is a feasible structure, which provides a reference direction for future device fabrication and testing.

## Data Availability

The data presented in this study are available from the corresponding author upon reasonable request.
